# Extensible and self-recoverable proteinaceous materials derived from scallop byssal thread

**DOI:** 10.1038/s41467-022-30415-3

**Published:** 2022-05-18

**Authors:** Xiaokang Zhang, Mengkui Cui, Shuoshuo Wang, Fei Han, Pingping Xu, Luyao Teng, Hang Zhao, Ping Wang, Guichu Yue, Yong Zhao, Guangfeng Liu, Ke Li, Jicong Zhang, Xiaoping Liang, Yingying Zhang, Zhiyuan Liu, Chao Zhong, Weizhi Liu

**Affiliations:** 1 Sars-Fang Centre, MOE Key Laboratory of Marine Genetics and Breeding, College of Marine Life Sciences, Ocean University of China, Qingdao, 266003 China; 2 Laboratory for Marine Biology and Biotechnology, Pilot National Laboratory for Marine Science and Technology, Qingdao, 266071 China; 3 Materials and Physical Biology Division, School of Physical Science and Technology, ShanghaiTech University, Shanghai, 201210 China; 4 Neural Engineering Centre, Shenzhen Institute of Advanced Technology, Chinese Academy of Sciences, Shenzhen, 518055 China; 5 School of Chemistry, Beihang University, Beijing, 100191 China; 6 National Center for Protein Science Shanghai, Shanghai Advanced Research Institute, Chinese Academy of Sciences, Shanghai, 201204 China; 7 Key Laboratory of Organic Optoelectronics and Molecular Engineering of the Ministry of Education, Department of Chemistry and Center for Nano and Micro Mechanics, Tsinghua University, Beijing, 100084 PR China; 8 Center for Materials Synthetic Biology, Shenzhen Institute of Synthetic Biology, Shenzhen Institutes of Advanced Technology, Chinese Academy of Sciences, Shenzhen, 518055 China; 9 CAS Key Laboratory of Quantitative Engineering Biology, Shenzhen Institute of Synthetic Biology, Shenzhen Institutes of Advanced Technology, Chinese Academy of Sciences, Shenzhen, 518055 China

**Keywords:** Biopolymers in vivo, Biosynthesis, Proteins, Biomaterials

## Abstract

Biologically derived and biologically inspired fibers with outstanding mechanical properties have found attractive technical applications across diverse fields. Despite recent advances, few fibers can simultaneously possess high-extensibility and self-recovery properties especially under wet conditions. Here, we report protein-based fibers made from recombinant scallop byssal proteins with outstanding extensibility and self-recovery properties. We initially investigated the mechanical properties of the native byssal thread taken from scallop *Chlamys farreri* and reveal its high extensibility (327 ± 32%) that outperforms most natural biological fibers. Combining transcriptome and proteomics, we select the most abundant scallop byssal protein type 5-2 (Sbp5-2) in the thread region, and produce a recombinant protein consisting of 7 tandem repeat motifs (rTRM7) of the Sbp5-2 protein. Applying an organic solvent-enabled drawing process, we produce bio-inspired extensible rTRM7 fiber with high-extensibility (234 ± 35%) and self-recovery capability in wet condition, recapitulating the hierarchical structure and mechanical properties of the native scallop byssal thread. We further show that the mechanical properties of rTRM7 fiber are highly regulated by hydrogen bonding and intermolecular crosslinking formed through disulfide bond and metal-carboxyl coordination. With its outstanding mechanical properties, rTRM7 fiber can also be seamlessly integrated with graphene to create motion sensors and electrophysiological signal transmission electrode.

## Abstract (web summary)

Bio-inspired materials are an intense area of study as researchers try to adapt biomaterials for other applications. Here, the authors report on the processing of protein materials derived from the byssal thread of scallops to create high-extensibility materials with self-recovery under wet conditions.

## Introduction

Nature has evolved the use of a serial of protein fibers, such as mussel byssal thread^1^, hagfish slime^2^, spider silk^3^, silkworm silk^4^, and rat tail tendon fiber^5^, with unique hierarchical structures^6,7^, functional domains^8–11^, and intermolecular crosslinks^12,13^, to achieve outstanding mechanical properties for specific functional roles. Inspired by these natural fibers, researchers have made important progress in the development of various protein-based fibers with outstanding mechanical properties, such as hagfish slime-inspired fibers with ultra-high stiffness^14^ and silkworm silk-inspired fibers with enhanced tougheness^15^, with enormous applications in tissue engineering^16^, extracellular matrix^17^, wearable device^18^, and other fields. From a sustainable viewpoint, those bio-derived fibers are excellent substitutes for chemically synthesized fibers^19,20^. Despite impressive strength, stiffness, and toughness, the application scope of existing biologically inspired materials is severely limited by their low extensibility and poor self-recovery capability, particularly in wet conditions. Biomaterials with high extensibility and self-recovery properties in wet physiological conditions are highly demanded for tissue engineering and medical care applications^21,22^, as they have the potential to support soft tissue repairing where a certain degree of elasticity, such as vocal folds^23^, human cartilage^24^, and muscle tissue^25^ is highly required. In addition, owing to their stretchability and low stiffness, they may find broad applications in wearable smart materials and flexible electronics, such as strain sensors with high sensitivity^26,27^.

**Fig. 1:**
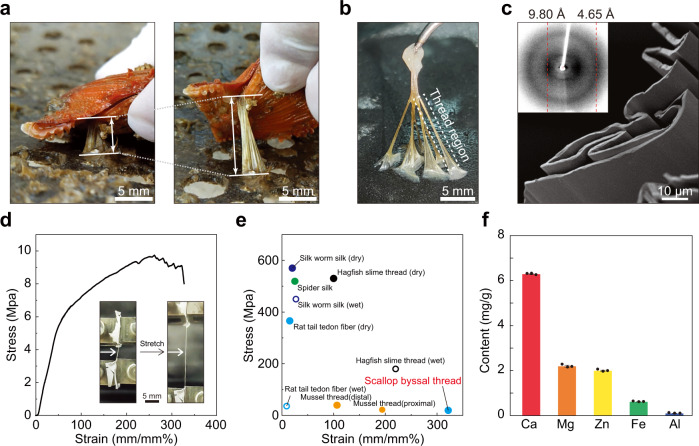
Structure and mechanical properties of the native scallop byssal thread. **a** Photographs of marine scallop adheres to a given substrate through a byssus with a bundle of threads before and after stretching. **b** Photographs of a complete byssus derived from scallop. The thread region of scallop byssus is marked in dotted box. **c** Morphological characterization of the microscopic structure of the byssal thread by SEM. The byssal thread is composed of films folded loosely and aligned along the axis of macroscopic byssus. The insert image refers to X-ray fiber diffraction pattern of the byssal thread. The byssal thread shows a typical diffraction pattern of cross-β strands, in which the meridional reflection is ~4.65 Å (corresponding to the inter-sheet distance within the same layer) and the equatorial reflection is ~9.80 Å (corresponding to the inter-sheet distance between adjacent layers). **d** A representative strain-stress curve of scallop byssal thread stretched in wet condition (relative humidity ~90% and tensile speed 0.2 mm/s). The byssal thread shows high extensibility reaching up to 327 ± 32%. The insert images show the byssal thread before and after stretching. **e** The extensibility comparison of scallop byssal thread and several biogenic threads derived from diverse of creatures underwater or on the earth. The scallop byssal thread shows the highest extensibility, which serves a benchmark for high extensible materials. **f** Quantitively analysis of metal elements in the byssal thread by ICP-MS. Among these polyvalent metals, calcium accounts for the largest proportion in thread region (55.75%). Data are presented as mean values ± SEM. *n* = 3 biologically independent experiments.

Scallop *Chlamys farreri* is able to firmly attach to a substrate using its byssus even in dynamic marine environment^28^. Scallop byssus possess high strength that can bear more than 100 times of its own body weight^29^. Another notable feature is that the byssal thread shows extraordinary extensibility, stretching more than twice of its original length (Fig. 1). Despite their attractive mechanical properties, little is known about the components and molecular assembly mechanisms of the scallop byssus. We previously identified the major components and distribution of scallop byssal proteins (Sbps) by combining transcriptomics and proteomics^29–32^. The byssus is composed of more than 20 different proteins and mucopolysaccharides^28,30,31,33^. Among them, several proteins including Sbp5-2 and Sbp4-1 were discovered as foot-specific proteins in the highly extensible region of the byssus thread, with Sbp5-2 being the most abundant component^30^.

The richness of Sbp5-2 in the byssal thread region raises an intriguing question whether this protein is responsible for the high-extensibility of the scallop byssus under wet condition. Here, we investigate the structure-mechanical properties relationship of the Sbp5-2 proteins across multiple length scales using a recombinant protein containing the eighth to fourteenth tandem repeats module (TRM8-14) of the Sbp5-2 protein as a model. We initially assess the hierarchical structure of the byssal thread derived from scallop *Chlamys farreri* and study its mechanical properties, including extensibility and self-recovery property. Next we achieve genetically engineered recombinant extensible protein 7 (rTRM7) fibers containing TRM8-14 of Sbp5-2 through a simple drawing process, recapitulating the hierarchical structure and mechanical properties of the scallop byssal thread. We further assess the possible inter- and intramolecular interactions including hydrogen bond, metal coordination bond, and disulfide bond in affecting the mechanical properties of the recombinant rTRM7 protein fibers. Finally, we illustrate a proof-of-concept demonstration of motion sensors and electrophysiological signal transmission electrode by embedding graphene in rTRM7 fibers through the above drawing process. We anticipate that our scallop-inspired rTRM7 fiber, which possesses high-extensibility and self-recovery capacity while retaining functionality in wet environment, would find broad applications in biomedical and industrial fields.

## Results

### Hierarchical structure and mechanical properties investigation of scallop byssal thread

Scallop *Chlamys farreri* anchor itself onto mineral substrates in the ocean through depositing a byssus with a bundle of threads. Interestingly, the thread region of the byssus exhibited remarkable extensibility (Fig. 1a). To understand the possible mechanisms, we took a fresh byssus from scallop and investigated the microscopic structure of the thread region (Fig. 1b). Morphological observation of the byssal thread by scanning electron microscope (SEM) clearly revealed that the thread is composed of smooth and loosely folded films that are aligned parallel to the byssus axis (Fig. 1c). Protein is the main component of the highly extensible thread, accounting for 81.89% of the total mass (Supplementary Table 1). To further study the secondary structure of the proteins in the byssal thread, we performed X-ray fiber diffraction and Fourier transform infrared (FTIR) spectroscopy on the thread region. The byssal thread showed a typical β-sheet diffraction pattern with meridional reflection at 4.65 Å (representing inter-sheet distance within β-sheet layers) and equatorial reflection at 9.80 Å (representing inter-sheet distance between β-sheet layers)^34^. Consistently, of all the secondary structures integrated from the amide I region of FTIR absorption spectrum, β-sheet structure accounts for the largest proportion, reaching 38.83 ± 1.75% (Supplementary Fig. 1 and Table 2).

We next conducted tensile test to study the mechanical properties of the byssal thread in high-moisture condition (relative humidity ~90%, tensile speed 0.2 mm/s) (Fig. 1d, Supplementary Movie 1). The resultant stress-strain curve revealed that the byssal thread showed high extensibility reaching 327 ± 32% of its original length, outperforming other types of natural fibers, including underwater mussel byssal thread^1^ and hagfish slime^2^, and spider silk^3,35^, silkworm silk^4^, and rat tail tendon fiber^5^ (Fig. 1e). Moreover, to study the self-recovery capability of the byssal thread, we performed cyclic tensile test by repeatedly stretching to 100% or 200% strain and returning to 0% strain for five consecutive cycles in wet condition (Supplementary Fig. 2). In these processes, the byssal thread showed a remarkable hysteresis, indicating effective energy dissipation upon deformation^36^. The dissipated energy substantially reduced in the second cycle, but remained constant in the following three cycles. The byssal thread showed little permanent deformation after cyclic stretching, indicating strong resistance to mechanical forces. These studies lead to a conclusion that scallops adopt high-extensible and self-recoverable byssal thread to optimize load distribution and energy dissipation^37^.

Shellfish usually leverages metal ions from seawater to construct their hierarchical structures and to strengthen the mechanical properties of their byssus. For example, mussel utilizes Fe^3+^ to enhance the stiffness of its byssus^38^, while pearl oyster adopts Ca^2+^ to stabilize the nanocavities within its byssus^39^. It is thus conceivable that scallop may adopt a similar mechanism for the mechanical benefits of its byssus thread. To test this hypothesis, we next applied inductively coupled plasma mass spectrometry (ICP-MS) to investigate the presence of polyvalent metal elements in the byssal thread of scallop. Quantitative analysis showed that Calcium was the most abundant element, accounting for 55.75% (Fig. 1f). Notably, after treatment of the byssal thread with ethylenediaminetetraacetic acid (EDTA) to remove possible polyvalent metal ions, we carried out similar tensile tests and found it maintained high extensibility, but the tensile strength decreased to 3.79 ± 0.65 MPa (compared to 9.88 ± 0.99 MPa for the non-EDTA treated sample). More intriguingly, after re-incubation in CaCl_2_ buffer (20 mM Tris-HCl, 10 mM CaCl_2_, pH 8.5), the byssal thread restored the tensile strength to 8.03 ± 1.22 MPa obviously arising from the reformation of metal-carboxy coordination bonds (Supplementary Fig. 3 and Table 3). These results highly implied that the calcium ions might coordinate with negatively charged groups (for example, carboxy groups) in the byssal thread proteins. Indeed, X-ray photoelectron spectroscopy (XPS) confirmed that the removal and recovery of Ca^2+^ in the EDTA-treated and Ca^2+^-recovery samples respectively: the Ca2*p* signal for the EDTA-treated byssal thread was much lower than that of the native and the Ca^2+^ recovery ones (Supplementary Fig. 4). Collectively, these results demonstrated that Ca^2+^ likely participates to form certain metal coordination bond that markedly enhanced the strength of scallop byssal thread.

### Scallop byssal thread-inspired protein-based fibers

**Fig. 2:**
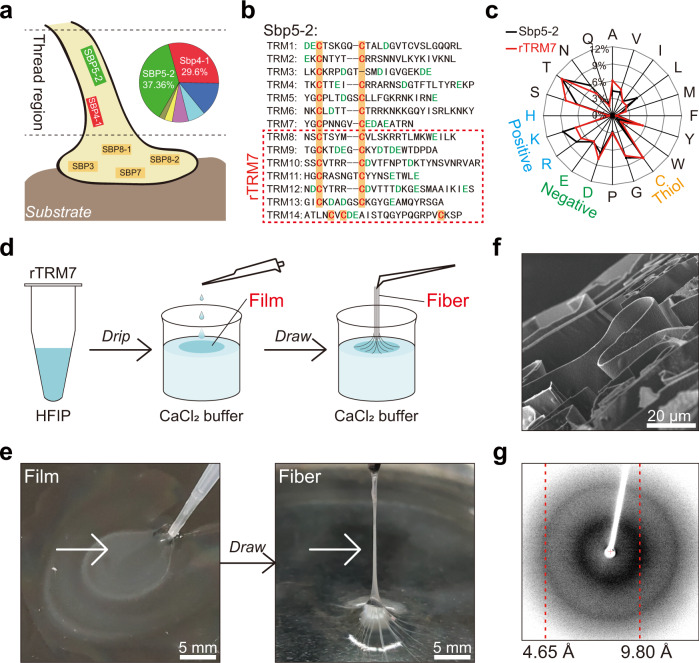
Highly extensible fibers made by recombinant Sbp5-2 analog proteins. **a** Schematic illustration of the spatial distribution of different proteins in scallop byssus. In the thread region, Sbp5-2 has been identified as the highest enriched protein (37.36%), among seven proteins. **b** Amino acid sequence of Sbp5-2 showing 14 highly identical flanking domains. Each TRM contains 2 Cys (C) (displayed in shade of orange), and several negative charged Asp (D) and/or Glu (E) (displayed in green font). rTRM7 was constructed rationally by fusing gene sequence of TRM8−14 marked in red dotted box. **c** The amino acid components of rTRM7 is compared with that of Sbp5-2. Amino acids composition and proportion of rTRM7 are both like that of Sbp5-2. Positively charged amino acids are shown in blue, negatively charged amino acids in green, and cysteine in orange. **d** Schematic illustration of fabricating rTRM7 fiber by drawing process. Freshly purified rTRM7 protein is firstly lyophilized and dissolved in HFIP at 200 mg/mL. Then, rTRM7 HFIP solution is casted into a film formed on CaCl_2_ buffer surface. Finally, rTRM7 fiber is picked up from solution surface by using forceps. **e** Photographs of rTRM7 film (left) and fiber (right) in drawing process corresponding to **d**. **f** SEM image of microscopic sectional view of rTRM7 fiber. **g** X-ray fiber diffraction pattern of rTRM7 fiber. rTRM7 fiber shows the same pattern of cross-β strand as scallop byssal thread, in which the meridional reflection is ~4.67 Å and the equatorial reflection is ~9.27 Å.

Intrigued by the high extensibility of scallop byssus and the fact that protein component is enriched in the thread region, we performed mass spectrometry to investigate proteins extracted from the thread region (Supplementary Fig. 5). Among them, Sbp5-2 (Gene ID: 30077.9)^31^, the highest expressed protein in the byssal thread accounting for 37.36 wt%, was hypothesized to be responsible for the high extensibility (Fig. 2a, Supplementary Fig. 6). Moreover, we obtained the amino acid sequence of Sbp5-2 from scallop genome, and confirmed it by rapid-amplification of cDNA ends (RACE) (Supplementary Table 4). The Sbp5-2 amino acid sequence can be divided into fourteen tandem repeat motifs (TRMs), each containing a Cys-X_n_-Cys pattern and several negatively charged residues (Fig. 2b). Notably, Cys accounts for 8.4% and negatively charged Asp and Glu accounts for 12.4% in Sbp5-2, implying the protein may contain both intermolecular disulfide bond and metal-carboxy coordination bonds. We thus reasonably speculated that the proteins enriched with TRMs are at least partially responsible for the high extensibility of the byssus. A further understanding of the structure-function relationship of TRMs would thus provide insights into the design of scallop byssal thread-inspired protein fiber.

Expression and purification of the full-length Sbp5-2 protein using *Escherichia coli* as a host turns out to be a challenging task possibly due to the high Cys content in the protein. To circumvent this difficulty, we rationally derived the gene sequence of the eighth to fourteenth tandem motifs from Sbp5-2 to construct a recombinant protein (rTRM7) (Fig. 2b), with the content of Cys and negatively charged Asp and Glu in rTRM7 closely matching that in Sbp5-2 (Fig. 2c). Indeed, with shortened sequence, rTRM7 could be successfully expressed as inclusion body in *E. coli* and could be directly purified as soluble protein following a denaturing protocol (Supplementary Fig. 7). By optimizing the protein expression and purification process, we could harvest ~120 mg protein per liter culture medium. Then, using an rTRM7 protein ink (lyophilized rTRM7 proteins dissolved in hexafluoro-2-propanol (HFIP) at 150 mg/mL)^40,41^, we developed a drawing process to fabricate rTRM7 fiber (Fig. 2d). Specifically, by initially casting rTRM7 protein ink into a thin and transparent free-standing film on CaCl_2_ buffer surface, we could slowly draw rTRM7 fibers from the surface of the flattened film using forceps. The as-prepared fibers were then immersed in CaCl_2_ buffer immediately (Fig. 2e). In the initial trials, we also attempted to produce fibers from the low-concentration rTRM7 (50 mg/mL), rTRM7 mutation (all Cys to Ser), and recombinant proteins containing less TRMs (≤6) and protein containing TRM1-7 of Sbp5-2. However, none of them could form films in the same condition (Supplementary Fig. 8). These results suggested that other than the specific protein sequence, factors including protein concentration, Cys residues and molecular length all affected the formation of thin-layer film and subsequent formation of thin fibers through the drawing process.

In order to identify the microscopic morphology and molecular structure of rTRM7 fiber, we performed SEM observations and X-ray fiber diffraction respectively. The SEM image results clearly revealed that rTRM7 fiber contained loosely folded smooth sheets that were aligned parallel to the fiber axis (Fig. 2f). Further, X-ray fiber diffraction pattern of rTRM7 fiber indicated a typical β-sheet structure with meridional reflection at 4.65 Å and equatorial reflection at 9.80 Å, like that of natural byssal thread (Fig. 2g). In addition, FTIR absorption spectrum revealed that of all the secondary structures in rTRM7 fiber, β-sheet constituted the largest proportion of 38.69 ± 7.86% (Supplementary Fig. 9 and Table 5). Collectively, these results suggested that the fabricated rTRM7 fiber recapitulated the hierarchical structure of natural scallop byssal thread.

### Mechanical properties and the underlying molecular interactions of rTRM7 fibers

**Fig. 3:**
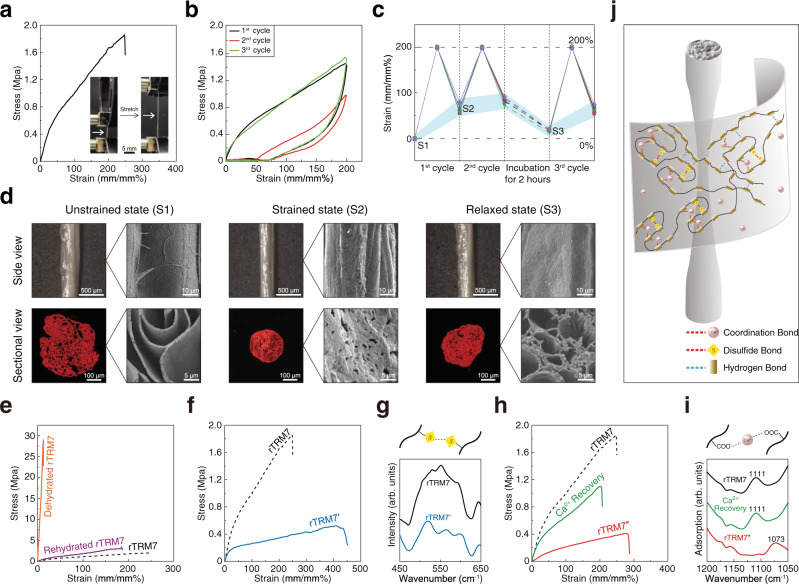
Mechanical properties investigation of rTRM7 fiber. **a** A typical strain-stress curve of the rTRM7 fiber stretched to rupture in wet condition. Inside images are an rTRM7 fiber before and after stretching. **b** Strain-stress curves of the rTRM7 fiber under cyclic tensile test at maximum strain of 200%. **c** Length change recording for rTRM7 fibers (*n* = 5) referring to initial state in a cyclic tensile test corresponding to the cyclic tensile test shown in figure **b**. S1 is unstrained state of fresh rTRM7 fiber. S2 is rTRM7 fiber after first stretch cycle. S3 is recovered rTRM7 fiber after a 2-h incubation in CaCl_2_ buffer. **d** Micro images (left) and SEM images (right) of side view (top) and sectional view (bottom) of the rTRM7 fibers in untrained, strained, and relaxed state corresponding to S1, S2, and S3 in figure **c**. **e** Representative strain-stress curves of rTRM7, dehydrated and rehydrated rTRM7 fibers. **f** Representative strain-stress curves of rTRM7 and rTRM7’ fiber stretched to rupture in wet condition. **g** Raman spectra of rTRM7 and rTRM7’ fibers corresponding to **f**. **h** Representative strain-stress curves of rTRM7, rTRM7”, and Ca^2+^ recovered rTRM7” fibers stretched to rupture in wet condition. **i** FTIR adsorption spectrum of rTRM7 fiber, rTRM7” fiber, and Ca^2+^ recovered rTRM7” fiber corresponding to **h**. The band of carboxy in rTRM7” fiber at 1073 cm^−1^ blue shifted to 1111 cm^−1^ indicated calcium-carboxy coordination was removed in rTRM7” and recovered in Ca^2+^ recovery rTRM7” fiber. arb. units, arbitrary unit. **j** Schematic illustration of lamellar structure and chemical bonds of recombinant protein fiber.

When stretching rTRM7 fiber with forceps under water, we observed a significant deformation reaching more than twice of its initial length (Supplementary Fig. 10). Therefore, we systematically studied the extensibility of rTRM7 fiber through tensile test. rTRM7 fiber showed high extensibility reaching 234 ± 35%, but relatively low tensile strength (1.21 ± 0.45 MPa) in wet condition, likely due to the water molecules trapped inside the fiber acting as plasticizer (Fig. 3a and Supplementary Movie 2). The statistics for mechanical measurements of ten individual fabricated fibers indicated the reproducibility of this method (Supplementary Fig. 11). Moreover, it was confirmed that the mechanical properties were not affected by the picking speed (Supplementary Fig. 12). When repeatedly stretching to 100% or 200% strain at 0.2 mm/s and finally restoring to 0 % strain for five cycles consecutively, rTRM7 fiber exhibited remarkable hysteresis and energy dissipation in the first cycle, but showed less pronounced behaviors in the following four cycles (Supplementary Fig. 13). We next investigated the self-recovery capability of rTRM7 fiber with programmed tensile test by firstly stretching to 200% stain at 0.2 mm/s and restoring to 0 % strain for three cycles at different time intervals (the second cycle test was executed immediately after the first cycle, but the third cycle test was carried out after incubating the stretched rTRM7 fiber in CaCl_2_ buffer for 2 h) (Fig. 3b). In the second cycle, the strain-stress curve of rTRM7 fiber did not restore to the first cycle and the dissipated energy reduced by ~80%. Whereas in the third cycle, rTRM7 fiber showed the same strain-stress curve as the first cycle with equal dissipated energy. These evidence indicated the fabricated rTRM7 fiber could recapitulate the high extensibility of the scallop byssal thread in wet condition, and also exhibited self-recovery capability.

Concurrently, we measured the length of rTRM7 fiber before and after each stretching in the aforementioned programmed tensile test (Fig. 3c). rTRM7 fiber was stretched longer after each tensile cycle test. However, after incubating in CaCl_2_ buffer for 2 h of relaxation, rTRM7 fiber almost restored to its original length. We then carried out further characterization with light microscopy and SEM to gain insights into the microscopic structure of rTRM7 fiber in unstrained, strained, and relaxed states (Fig. 3d). CdSeS@ZnS quantum dots (QDs), which could bind rTRM7 molecules specifically via His-tag affinity, was utilized to visualize the cross section of rTRM7 fiber. After stretching, microscopic images showed that the strained rTRM7 fiber became thinner and denser than in unstrained state. In parallel, SEM images revealed that the films of the strained rTRM7 fiber became highly compacted and the original smooth surfaces turned into wrinkled surfaces. After incubating in CaCl_2_ buffer for 2 h, the surface of rTRM7 fiber, as revealed by both microscopic and SEM imaging, became smooth again and the internal aperture was enlarged again. Based on these observations, we concluded that rTRM7 fiber possessed self-recovery capability that can restore both the energy dissipation and microscopic structure within 2 h of incubation in CaCl_2_ buffer.

Intermolecular interactions have always been considered as one of the key factors affecting the mechanical properties of materials^38,41^. Animal threads such as the thread in a spider web are enriched with hydrogen bonds that heavily affect their mechanical properties^42,43^. Thus, we conjecture that hydrogen bond might affect the extensibility and tensile strength of the fabricated rTRM7 fiber. When rTRM7 fiber was dried at room temperature (relative humidity ~50%) for 10 min, the extensibility sharply decreased to 11.3 ± 2.9% and the tensile strength increased to 36 ± 14 MPa (Fig. 3e). After incubating the dried fibers in CaCl_2_ buffer for 10 min, rTRM7 fiber restored the extensibility to 228 ± 34% and the tensile strength to 4.4 ± 1 MPa, implying the hydrogen bond could quickly restore in the hydrated fibers (Fig. 3e). Conceivably, the hydration state is essential to maintain the hydrogen bond within rTRM7 fiber, which in turn contributes to the extensibility but reduces the tensile strength of rTRM7 fiber. In addition, the fiber still maintained excellent mechanical properties even dispersed in the PBS buffer at 37 °C for 3 days (Supplementary Fig. 14). However, the addition of protease in the same PBS buffer could result in observed degradation of fibers after incubation for 24 h (Supplementary Fig. 15), implying the intrinsic biodegradation of the rTRM7 fiber under physiological condition.

Other noticeable features of rTRM7 fiber include the presence of Cys in the proteins that might form disulfide bond, and the negatively charged Asp and Glu that can coordinate with metal ions. To rule out possible influence of the formation of disulfide bond on mechanical properties, we produced identical rTRM7 fiber (referred as rTRM7’ fiber by drawing from CaCl_2_ buffer with dithiothreitol (DTT) (20 mM Tris-HCl, 10 mM CaCl_2_, 100 mM DTT, pH 8.5)), a condition that disrupts or disfavors the formation of disulfide bond. The extensibility of rTRM7’ fiber increased to 475 ± 16% and the tensile strength decreased to 0.42 ± 0.11 MPa (Fig. 3f). The presence of disulfide bond in rTRM7 and rTRM7’ fibers was next assessed by Raman spectroscopy: the characteristic peak ranging from 495 cm^−1^ to 635 cm^−1^ confirmed that most of the disulfide bonds were eliminated in rTRM7’ fiber (Fig. 3g)^44^.

Subsequently, to investigate the mechanical properties changes caused by metal-carboxy coordination, we prepared and tested three groups of fibers: rTRM7 fiber as control, rTRM7” fiber without metal-carboxy coordination drawn from buffer with EDTA (20 mM Tris-HCl, 100 mM EDTA, pH 8.5), and Ca^2+^ recovered rTRM7” fiber obtained by incubating fiber in CaCl_2_ buffer for 2 h (Fig. 3h). The extensibility of rTRM7” fiber increased to 300 ± 43%, but the tensile strength reduced to 0.43 ± 0.12 MPa, whereas Ca^2+^ recovered rTRM7” fiber restored the extensibility to 221 ± 28% and the tensile strength to 0.79 ± 0.30 MPa. The calcium-carboxy coordination bond in the fibers were monitored by FTIR^45^. Compared to rTRM7” fiber, the band of carboxy at ~1073 cm^−1^ blue shifted to ~1111 cm^−1^ implying that calcium-carboxy coordination was missing in rTRM7” fibers and recovered in Ca^2+^ recovered rTRM7” fiber (Fig. 3i)^46^. The presence of Calcium elements in these fibers were also confirmed by XPS revealing that Ca2*p* signal appeared in rTRM7 fiber and Ca^2+^-recovered rTRM7” fiber, but not in rTRM7” fiber (Supplementary Fig. 16). In parallel, secondary structure analysis from the Amide I region of FTIR spectra revealed that β-sheet content in rTRM7” fiber was less than in Ca^2+^-recovered rTRM7” fiber, and the β-sheet content in Ca^2+^-recovered rTRM7” fiber restored to that in rTRM7 fiber (Supplementary Fig. 17). Therefore, Ca^2+^ promoted β-sheet formation implied a potential way to enhance tensile strength.

Furthermore, in cyclic tensile test stretching to 100% strain, rTRM7’ and rTRM7” fibers both exhibited lower energy dissipation capacity than rTRM7 fiber (Supplementary Fig. 18). Then in programmed tensile test, we incubated the two-cycle stretched rTRM7’ and rTRM7” fibers in CaCl_2_ buffer for 2 h to reform the disulfide bond and metal-carboxy coordination. In the third cycle test, rTRM7’ and rTRM7” fibers both restored the energy dissipation capacity and restored the tensile strength, which were both much higher than in the third cycle (Supplementary Fig. 19). All these results indicated that dynamic disulfide bond and metal-carboxy coordination in rTRM7 fiber contributed to self-recovery and energy dissipation, and enhanced the tensile strength but restricted the extensibility, through restricted film shearing, controlled slippage, and stress transfer, although direct experimental evidence for these behaviors in rTRM7 fiber was lacking (Fig. 3j)^47^.

### Functional rTRM7 fiber/graphene hybrid wearable devices

**Fig. 4:**
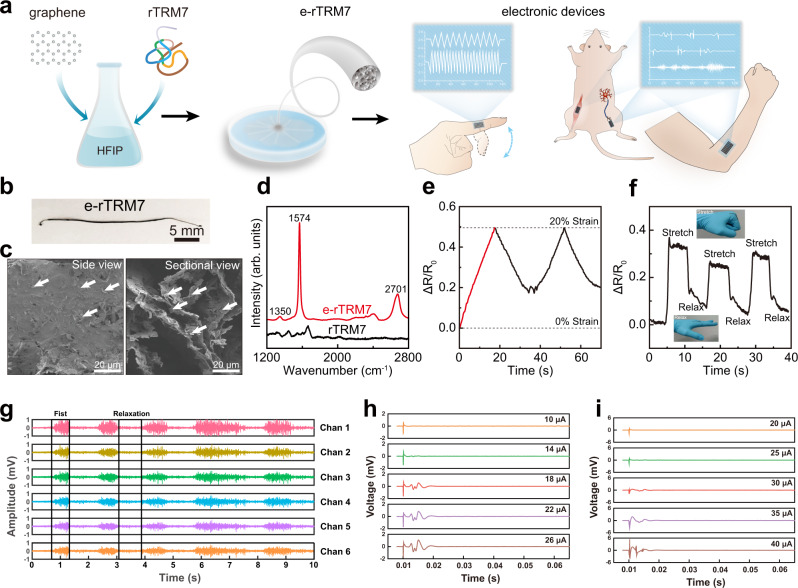
Application of conductive e-rTRM7 fiber as motion sensor. **a** Schematic illustration of fabricating electronic rTRM7 (e-rTRM7) fiber and its applications in wearable and implantable electrodes. **b** Photograph of e-rTRM7 fiber. **c** SEM images of side view and sectional view of the e-rTRM7 fiber showing graphene flakes are randomly embedded in the films of e-rTRM7 fiber. The white arrows point to the graphene flakes. **d** Raman spectrum of e-rTRM7 and rTRM7 fibers. Typical graphene peaks at 1350, 1574, 2701 cm^−^^1^ are appeared in e-rTRM7 fiber, while not in rTRM7 fiber. A.U., arbitrary unit. **e** Resistance change of e-rTRM7 fiber over time under cycle loading-unloading stretching. e-rTRM7 fiber is stretched to 20% strain and relaxed to 0% strain in two continuous cycles. The resistance is positively related to the strain of e-rTRM7 fiber (red part). **f** e-rTRM7 fiber applied to knuckle as a motion sensor. Resistance changes of e-rTRM7 fiber over time when hand fist for 6 s and relax for 6 s repeatedly corresponding to the picture. **g** EMG signals produced by the fist and relaxation. **h**, **i** The in vivo action potential signals from tibialis anterior muscle and tibial nerve induced by the increased stimulating current on common peroneal nerve of the rat.

In recent years, motion sensor, which can be noninvasively mounted on human body, have been intensively studied by researchers in the field of wearable electronics^48,49^. However, preparing high-performance motion sensor with high extensibility and high sensitivity in wet environment is still challenging^50^. Here, the electronic properties of rTRM7 fiber were carried out in wet environment showing that the resistance of rTRM7 periodically changed with cyclic stretching, but the performance was non-reproducible (Supplementary Fig. 20). The combination of biomacromolecule and carbon nanomaterials provides a feasible solution for stable and wearable sensors^51,52^. Existing conventional top-down approaches to obtain conductive materials embedded within such motion sensors often requires multi-step processes that are both time-consuming and costly^53,54^. Hence, we designed and fabricated electronic rTRM7 (e-rTRM7) fiber by introducing conductive graphene during the drawing process. In particular, e-rTRM7 fiber was obtained through a similar TRM7 film drawing process using a mixed solution with graphene flakes dispersed in an rTRM7 HFIP solution (150 mg/mL rTRM7 and 2% graphene) (Fig. 4a-b). Combined with the certain study described previously^55,56^ and morphological observation by SEM imaging revealed that graphene flakes were randomly but evenly embedded within the films of e-rTRM7 fiber (indicated by white arrow in Fig. 4c). The presence of graphene was also confirmed by Raman spectroscopy (Fig. 4d). Compared with rTRM7 fiber, three typical peaks attributing to graphene at ~1350 (D band), ~1574 (G band), and ~2701 (G’ band) cm^−1^ appeared in the Raman spectrum of e-rTRM7 fiber^57^. We next conducted tensile test to investigate the mechanical properties of e-rTRM7 fiber, which showed extensibility of 92 ± 11 % and tensile strength of 0.38 ± 0.09 MPa in wet condition. Noticing that an obvious decrease in mechanical properties of protein fibers occurred when incorporated with graphene, thus we next performed morphological comparison of the structures for the rTRM7 and e-rTRM7 fiber with SEM imaging (Figs. 3d and 4c). The SEM results of e-rTRM7 fibers showed that graphene flasks evenly dispersed in the sample and seemed to disrupt the complete lamellar structures of the protein fibers, as the lamellar structures became less dense after embedding graphene. The partial disruption of the lamellar structure may weaken the interaction between protein molecules in the fibers, resulting in a substantial decrease in the mechanical properties of the fibers. However, the extensibility of e-rTRM7 fibers still outperforms most of the graphene embedded fibers reported so far^58–61^.

The well-dispersed graphene flakes in the films of rTRM7 form an electrically conductive path that is responsive to environmental changes, such as strain and external force, endowing the e-rTRM7 fiber with high sensitivity to stimuli. We explored the resistance change of e-rTRM7 fiber during cyclic stretching to 20% strain and backing to 0% strain at a speed of 0.2 mm/s in wet condition (Fig. 4e). The red part indicated the resistance of e-rTRM7 was positively related to the strain of e-rTRM7 fiber with high sensitivity (gauge factor: ~2.5). In the relaxation stage, the resistance of e-rTRM7 fiber decreased but not restored to the starting value due to the slow recovery rate of fiber deformation. In addition, e-rTRM7 fiber exhibited consistent resistance change in continuous loading-unloading test (Supplementary Fig. 21). Cytotoxicity assays showed that e-rTRM7 fibers exhibited good cytocompatibility (Supplementary Fig. 22). To demonstrate e-rTRM7 fiber’s practical application as a wearable strain sensor, it was directly mounted on a knuckle to monitor finger activity (Fig. 4f). The real-time monitoring showed the resistance of e-rTRM7 fiber increased when making a fist and recovered when relaxing (Fig. 4f). Based on all these observations, e-rTRM7 fiber combining the mechanical properties of rTRM7 fiber and the electrical properties of graphene, with high extensibility and high sensitivity, would serve as the next-generation wearable electronic system in diverse fields such as medicine, entertainment, and sport industry.

The graphene decorated rTRM7 fiber electrodes can further be used for skin electromyography (EMG) and in vivo compound muscle action potentials detection. As shown in Supplementary Fig. 23 and Movie 3, six fiber electrodes were placed on the forearm (outer sider) of a volunteer. When the hand made a fist and relaxation, the wrist flexors contracted and generated surface EMS signals. Different gestures by finger performing flexion and extension (Fig. 4g and Supplementary Fig. 24) were also demonstrated to illustrating the graphene decorated rTRM7 fiber can be well used for skin EMG detection function. The amplitude of the signal peaks among those six electrodes showed the consistency of the prepared fibers. Further, the detection of skin EMG signals for muscle motion suggests its potential application in the human-machine interfaces. In addition, in vivo compound muscle action potential signals detection was also performed (Fig. 4h, i and Supplementary Movie 4). The stimulating signal was introduced through the common peroneal nerve of the rat. Accordingly, the action potential signals from tibialis anterior muscle and tibial nerve were recorded respectively. By applying the increased stimulating current (10-40 μA), the peaks of the action potential signals became more distinct. The in vitro and in vivo physiological signals detection functions combined with the excellent biocompatibility suggest the ability of skin-attachable and implantable electrode for bio-interactive applications.

## Discussion

In this work, we investigated the mechanical properties and hierarchical structure of the naturally occurring scallop byssal thread, and demonstrated TRMs in Sbp5-2 were responsible for the high extensibility and self-recovery capability. We next genetically constructed a recombinant protein consisting of 7 tandem repeat motifs (rTRM7) of the Sbp5-2 protein and expressed the protein in *E. coli* host with improved production yield for scalable application. rTRM7 fiber was prepared by a simple drawing process, recapitulating the hierarchical structure and mechanical properties of scallop byssal thread, with impressive extensibility and self-recovery capability.

Notably, we observed that intermolecular crosslinks, mainly contributed by hydrogen bond, metal-carboxyl coordination, and disulfide bond, had a significant influence on the extensibility and self-recovery capacity of rTRM7 fiber, suggesting an effective way to modulate it by modifying the formation of intermolecular crosslinks. Moreover, by embedding graphene into rTRM7 fiber, we achieved high performance e-rTRM7 fiber and established a proof-of-concept application as motion sensor and electrophysiological signal transmission electrode to monitor human body activities.

Our studies shed light on the molecular mechanism underlying the extensibility of scallop byssal thread and can guide new design to construct bio-inspired protein-based fibrous materials from sustainable protein feedstocks, especially for applications in wet environment. To tap its full potential, further work will be needed to probe the detailed intermolecular assembly mode for TRMs. On the application side, the fibrous materials based on TRM with outstanding mechanical performance represents a new class of high extensible materials for diverse application scenario. New TRMs-based proteins with additional functionalities through genetic engineering may further expand their application scope in both industrial and medical settings.

## Methods

### Scallop byssal thread protein collection

*Chlamys farreri* scallop was purchased from Qingdao seafood market and cultured in laboratory conditions (~19 °C) for 24 h to secrete byssus. The byssal thread was harvested and washed with amounts of deionized water. Then, we extracted the soluble proteins by following the methods described in previous papers^30,62^. A clean blade was used to cut the thread part of the scallop byssus for later usage. The samples were then dissolved using the extraction buffer (6 M guanidine hydrochloride, 5% acetic acid, 2 mM EDTA, 10 mM DTT) after grinding evenly in liquid nitrogen. After incubation at 37 °C for 2 h, the sample was centrifuged at 12,000 g at 4 °C for 25 min. The resulting supernatant was first dialyzed against 0.5 % acetic acid solution twice and then against deionized water once. The soluble protein components were freeze-dried and preserved for later usage. The soluble proteins were separated by SDS-PAGE and the main proteins in brightest bands marked in red boxes were collected for further study. Data is presented in Supplementary Fig. 5.

### MS

The main bands shown as red rectangles in Fig S4 were cut with a clean blade for mass spectrometry. MS spectra were acquired by Q-Exactive mass spectrometer (Thermo Scientific) in positive mode over a range of 300 to 2000 m/z. The strongest ten signals were selected and analyzed by Proteome Discoverer 1.4 (Thermo Scientific). To estimate protein abundance, we searched the fragmentation spectra against the C*hlamys* farreri full-protein database by Proteome Discoverer with Mascot search engine. Data are presented in Fig. 2a and Supplementary Fig. 6.

### ICP-MS

The byssal thread was lyophilized and digested in 5 mL nitric acid for 2 h, and then diluted to 50 mL with deionized water. The sample solution was analyzed by Agilent 7500CE ICP-MS (Agilent Technologies Co. Ltd, USA). Commercial Sc, Li, Y, Ge, In, Tb, and Bi samples at 1.0 mg/L were selected as a mixed internal standard. Data is presented in Fig. 1f.

### Amino acid analysis

13.73 mg of dried byssal thread was accurately weighed and put into a glass tube, and 6 M HCl was added to the tube and sealing. The byssal thread was hydrolyzed for 24 h in a 110 ± 1 °C thermostatic drying oven. After the hydrolysate cools, the mixture was poured into a crucible and dried in a water bath after cooling. The remaining sample was dissolved by adding 0.01 M HCl and placed at room temperature for 4 h to convert cysteine to cystine, hydrolyzed protein sample was finally dissolved in 0.02 M HCl and injected into the amino acid analyzer (Hitachi L-8900 automatic amino acid analyzer) for analysis. Data are presented in Supplementary Table 1.

### Plasmid construction

The amino acid sequence of Sbp5-2 was confirmed by rapid-amplification of cDNA ends (RACE). rTRM7, rTRM6, and rTRM7 mutation (all Cys to Lys) genes were cloned from Sbp5-2 by PCR. The target genes were digested by BamHI and XhoI restriction enzymes (Takara) at 25 °C for 30 min, and inserted into pET32a by T4 Ligase at 37 °C for 30 min. The recombinant plasmids were then transformed into DH5α *Escherichia coli* competent cells (Novagen) respectively. All constructed plasmids were sequenced and verified by Sangon. Primers utilized in plasmids construction are listed in Supplementary Table 4.

### Protein expression and purification

The recombinant plasmids containing rTRM7, rTRM6, and rTRM7 mutation were transformed into BL21 (DE3) *Escherichia coli* competent cells (Novagen) individually. The strains were fermented in LB medium containing 50 mg/L kanamycin to OD_600_ of 0.8 at 37 °C. Protein expression was then induced with 0.2 mM Isopropyl-beta-D-thiogalactopyranoside (IPTG) at 37 °C for 10 h. The expressed bacteria were collected by centrifugation at 3,000 g at 4 °C for 30 min, and the pellets were suspended in phosphate buffer and lysed under sonication for 35 min (5 s / 10 s) by ultrasonic cell disruptor (JY 92-IIN Scientz). The insoluble inclusion body was collected by centrifugation at 12,000 g for 10 min, purified twice with 50 mL PBS, and then purified twice with 50 mL 1 M urea buffer (20 mM Tris-HCl, 1 mM EDTA, 1% Triton-114, 1% glycerin, pH 8.5). The purified inclusion body was then dissolved in 8 M urea buffer (20 mM Tris-HCl, 10 mM DTT, pH 8.5) and dialyzed in dialysate buffer (20 mM Tris-HCl, 1 mM DTT, pH 8.5) for protein refolding. The purified protein was identified by SDS-PAGE and lyophilized for further study. Data is presented in Supplementary Fig. 7.

### Fiber preparation

The lyophilized recombinant proteins were dissolved in HFIP at a final concentration of 150 mg/mL. Five microlitre of the protein HFIP solution was then dropped onto the surface of Tris-HCl buffer in a petri dish, and then the self-assembling films forming on buffer surface were picked up with forceps and drawn into fiber (Supplementary Movie 5). In addition, to evaluate the pick speed influences on the fibers, we fabricated the protein fibers using a typical Dip Coater (MHY-08093, Beijing Meihuayi Technology Co., LTD.) at different speed (50 mm/min, 100 mm/min, and 200 mm/min) (Supplementary Movie 6). The freshly made fiber was then incubated in CaCl_2_ buffer for 24 h for full maturation. Recombinant protein fibers were washed with a large amount of deionized water and lyophilized for further characterization. Data are presented in Fig. 2d, Supplementary Fig. 8.

### e-rTRM7 fiber preparation

Graphene ink (KNG^®^-G2) was purchased from Knano Graphene Technology Co., Ltd. China. The graphene powder was dissolved and well dispersed in rTRM7 HFIP solution at molecular weight of 2% MW. Then the e-rTRM7 fiber was prepared following the above fiber preparation process. Data are presented in Fig. 4a, b.

### Tensile testing

Scallop byssal thread and recombinant protein fibers were carefully adhered to cardstocks with initial test length of 5 mm. The mechanical properties of fibrous materials were measured by Electromechanical Universal Testing Machine (MTS Systems Co., Ltd.) equipped with a tensile speed of 0.2 mm/s. A 10 N load cell (MTS Systems Co., Ltd.) was applied to measure the force. All tests were carried out in wet condition (relative humidity ~90%). The reproducibility of fabricated fibers was evaluated by measuring the mechanical properties of ten fibers. The strain was calculated as the change in length divided by the initial test length, and the stress was calculated as the force divided by the cross-sectional area. The diameter of fibers were determined through optical microscope (VHM 3201) and cross-sectional area of the protein fiber that we used was based on the geometric area (including the inner space between protein layers). The fiber lengths were also recorded before and after stretching. Data are presented in Figs. 1d, 3a, b, c, e, f, h, Supplementary Figs. 3, 10, 11, 12, Supplementary Movie 1 and 2.

### Self-recovery test

To test the self-recovery properties of the recombinant fibers, the fibers were stretched to 200% strain and then returned to 0% strain. After two cycles of continuous stretching, the fibers were removed from the fixture and incubated in the buffer (20 mM Tris-HCl, 10 mM CaCl_2_, pH 8.5) for different times (0.5 h, 1 h, and 2 h), and then the incubated fibers were stretched for the third cycle. In order to fully restore the mechanical properties to the initial level, we incubate the fiber in the same buffer for 2 h in all experiments. We also performed multiple recovery experiments on our rTRM7 fibers (after each stretch cycle, the fibers were incubated in buffer for 2 h, and the recovery test was performed at least ten times). To further investigate the self-recovery ability of byssal thread and recombinant protein fibers, we subject five successive loading-unloading cycles at strain of 100% and 200%. Data are presented in Supplementary Figs. 2, 13, 18, 19, 25, and 26.

### Stability and enzyme degradation evaluation

The fabricated protein fibers were respectively immersed in PBS buffer and PBS solution with 3.5 U/mL protease XIV (Sigma Aldrich), followed by incubation of at 37 °C in a sterile environment. The mechanical properties of the fibers were measured every 24 h. Data are presented in Supplementary Figs. 14 and 15.

### Resistance measurement

The resistance of the e-rTRM7 fiber was recorded by digital multimeter (Keithley 2400) with a resolution of 1 s. In order to monitor hand movement, we adhered the e-rTRM7 fiber to the knuckle and measured the resistance change while making a fisting. Data are presented in Fig. 4e, f, Supplementary Figs. 20 and 21.

### Biopotential signal extraction

The EMG signals were acquired by placing six graphene decorated rTRM7 fiber electrodes on the forearm and a reference electrode on the elbow. The front end of the fiber electrodes was attached with skin, while the trailing end was sandwiched by two copper foil, which was connected to the signal-out system. After that, those electrodes were connected to a self-designed signal-recording setup processed with a band-reject filter (48–52 Hz). All of the fibrous materials in the tests were tested in high humidity conditions and in humoral environments. The EMG signals were analyzed using the MATLAB (2019b) envelope function. For the in vivo experiment, the stimulating electrode was put onto common peroneal nerve of male SD rats. The graphene decorated rTRM7 fibers were put onto tibialis anterior muscle and tibial nerve. And the compound muscle action potentials on tibialis anterior muscle and tibial nerve were recorded using Plexon equipment. Data are presented in Fig. 4g, h, i, Supplementary Figs. 23, 24, Movie 3 and 4.

### SEM imaging

SEM images were collected using a JSM 7800 SEM with 2 kV accelerating voltage. For sectional view, the samples were cut vertically by a clean blade. Data are presented in Figs. 1c, 2f, 3d, and 4c.

### X-ray fiber diffraction

The X-ray fiber diffraction data were collected using a Rigaku MicroMax 007 x-ray generator and an R-AXIS IV + + area detector. Fibrous samples were put into glass capillary tubes and mounted on the sample stage. Data are presented in Figs. 1c and 2g.

### XPS

XPS was executed on an ESCALAB 250Xi (Thermo Fisher Scientific) with a monochromatic Al-Kα line (1486.6 eV). Element signals of samples were collected with a step width of 0.01 eV. Data are presented in Supplementary Figs. 4 and 16.

### FTIR spectroscopy

FTIR spectra were acquired by Nicolet iN10 infrared spectrometer (Thermo Scientific) in attenuated total reflection mode (ATR) from 4000 to 400 cm^−1^ with a nominal resolution of 4 cm^−1^. Baseline subtraction and peak fitting were performed with Omnic 8.2 (Thermo Fisher Scientific Inc., Waltham, MA, USA). Data are presented in Fig. 3i, Supplementary Figs. 1, 9 and 17.

### Raman spectroscopy

Raman spectra were obtained by Labram HR800 (Horiba, France) spectrometer with 532 nm laser excitation (10 mW power) from 200 to 3000 cm^−1^. The acquisition time of each sample spectrum was 30 s, and the accumulation was 20 times. The Raman system was calibrated with a silicon slice with the characteristic peak at 520 cm^−1^. Data are presented in Figs. 3g and 4d.

### In vitro cytotoxicity assay

Microtitration (MTT) assay was used to test the cytotoxicity of the fabricated fibers by assessing the viability of Rat L929 cells in the presence of the e-rTRM7 fibers following the guideline from ISO 10993-5. The fabricated e-rTRM7 fibers were sterilized by submersing the samples in 75% (v/v) ethanol and disinfected with ultraviolet light for 60 min, then washed with sterilized PBS solution three times for 90 min. The fiber extract was prepared by incubating the sterilized e-rTRM7 fibers with 5 ml culture medium prepared with DMEM (addition of 10% (v/v) FBS and 0.5% (v/v) Penicillin–Streptomycin) and incubated for 24 h (37 °C, 5% CO_2_ and 95% air). 96 well microplates containing culture medium or fiber extract were used to grow cells. Each of the microplate wells contained around 1 × 10^4^ cells and cultured in an incubator with 5% CO_2_ at 37 °C. Subsequently, after culturing for 1 day, 3 days, and 5 days separately, 20 μL MTT solution (5 mg/mL) was added to each well. The culture medium was taken away after 4 h incubation. 100 μL of DMSO was applied to each well and detect UV absorption intensity at 570 nm wavelength after incubation for another 5 min. Data are presented in Supplementary Fig. 22.

### Statistics and reproducibility

All experiments were repeated three times independently with similar results.

## Supplementary information

Supplementary Information

Description of Additional Supplementary Files

Supplementary Movie 1

Supplementary Movie 2

Supplementary Movie 3

Supplementary Movie 4

Supplementary Movie 5

Supplementary Movie 6

## Data Availability

The datasets generated in this study are provided in the Supplementary Information/Source data file. Source data are provided with this paper and all Figures and Supplementary Figures/Tables in the associated source data file are available. Source data is available for Figs. 1d–f, 2a–c, 3a–c, 3e–i and 4d–i and Supplementary Figs. 1, 2, 3, 4, 6, 7, 9, 11, 12, 13, 14, 16, 17, 18, 19, 20, 21, 22, 24, 25, and 26 in the associated source data file. Gene sequences are available at the scallop genome website (http://mgbase.qnlm.ac/home). Source data are provided with this paper.

## Source data

Source Data

## References

[1] Harrington MJ, Waite JH (2009). How nature modulates a fiber’s mechanical properties: mechanically distinct fibers drawn from natural mesogenic block copolymer variants. Adv. Mater..

[2] Fudge DS, Gosline JM (2004). Molecular design of the alpha-keratin composite: insights from a matrix-free model, hagfish slime threads. Proc. Biol. Sci..

[3] Plaza GR (2012). Relationship between microstructure and mechanical properties in spider silk fibers: identification of two regimes in the microstructural changes. Soft Matter.

[4] Plaza GR (2008). Effect of water on *Bombyx mori* regenerated silk fibers and its application in modifying their mechanical properties. J. Appl. Polym. Sci..

[5] Kato YP (1989). Mechanical properties of collagen fibres: a comparison of reconstituted and rat tail tendon fibres. Biomaterials.

[6] Guo C (2018). Structural comparison of various silkworm silks: an insight into the structure-property relationship. Biomacromolecules.

[7] Harrington MJ, Jehle F, Priemel T (2018). Mussel byssus structure-function and fabrication as inspiration for biotechnological production of advanced materials. Biotechnol. J..

[8] Rising A (2007). Major ampullate spidroins from *Euprosthenops australis:* multiplicity at protein, mRNA and gene levels. Insect Mol. Biol..

[9] Suzuki Y, Yamazaki T, Aoki A, Shindo H, Asakura T (2014). NMR study of the structures of repeated sequences, GAGXGA (X = S, Y, V), in Bombyx mori liquid silk. Biomacromolecules.

[10] Coyne KJ, Qin XX, Waite JH (1997). Extensible collagen in mussel byssus: a natural block copolymer. Science.

[11] Yang YJ, Jung D, Yang B, Hwang BH, Cha HJ (2014). Aquatic proteins with repetitive motifs provide insights to bioengineering of novel biomaterials. Biotechnol. J..

[12] Degtyar E, Harrington MJ, Politi Y, Fratzl P (2014). The mechanical role of metal ions in biogenic protein-based materials. Angew. Chem. Int. Ed..

[13] Ashton NN, Stewart RJ (2019). Aquatic caddisworm silk is solidified by environmental metal ions during the natural fiber-spinning process. FASEB J..

[14] Fu J (2017). Artificial hagfish protein fibers with ultra-high and tunable stiffness. Nanoscale.

[15] Mohammadi P (2019). Biomimetic composites with enhanced toughening using silk-inspired triblock proteins and aligned nanocellulose reinforcements. Sci. Adv..

[16] Green JJ, Elisseeff JH (2016). Mimicking biological functionality with polymers for biomedical applications. Nature.

[17] Wen C, Kang H, Shih YR, Hwang Y, Varghese S (2016). In vivo comparison of biomineralized scaffold-directed osteogenic differentiation of human embryonic and mesenchymal stem cells. Drug Deliv. Transl. Res..

[18] Chang H (2018). Ultrasensitive and highly stable resistive pressure sensors with biomaterial-incorporated interfacial layers for wearable health-monitoring and human-machine interfaces. ACS Appl. Mater. interfaces.

[19] Aigner TB, DeSimone E, Scheibel T (2018). Biomedical applications of recombinant silk-based materials. Adv. Mater..

[20] Ma D, Wang Y, Dai W (2018). Silk fibroin-based biomaterials for musculoskeletal tissue engineering. Mater. Sci. Eng. C.-Mater. Biol. Appl..

[21] Jing H (2021). Ultra-stretchable, self-recovering, self-healing cationic guar gum/poly(stearyl methacrylate-co-acrylic acid) hydrogels. Carbohydr. Polym..

[22] Aghaei-Ghareh-Bolagh B, Mithieux SM, Weiss AS (2016). Elastic proteins and elastomeric protein alloys. Curr. Opin. Biotechnol..

[23] Linqing L, Sean T, Clifton RJ, Xinqiao J, Kiick KL (2011). Tunable mechanical stability and deformation response of a resilin-based elastomer. Biomacromolecules.

[24] Renner JN, Cherry KM, Su RS, Liu JC (2012). Characterization of resilin-based materials for tissue engineering applications. Biomacromolecules.

[25] Lv S (2010). Designed biomaterials to mimic the mechanical properties of muscles. Nature.

[26] Boland CS, Khan U, Benameur H, Coleman JN (2017). Surface coatings of silver nanowires lead to effective, high conductivity, high-strain, ultrathin sensors. Nanoscale.

[27] Zheng Y (2020). High-performance wearable strain sensor based on graphene/cotton fabric with high durability and low detection limit. ACS Appl. Mater. interfaces.

[28] Caddy JF (1972). Progressive loss of byssus attachment with size in the sea scallop, Placopecten magellanicus (Gmelin). J. Exp. Mar. Biol. Ecol..

[29] Xu P (2018). The discovered chimeric protein plays the cohesive role to maintain scallop byssal root structural integrity. Sci. Rep..

[30] Miao Y (2015). Integration of transcriptomic and proteomic approaches provides a core set of genes for understanding of scallop attachment. Mar. Biotechnol..

[31] Li Y (2017). Scallop genome reveals molecular adaptations to semi-sessile life and neurotoxins. Nat. Commun..

[32] Zhang L (2019). Identification and characterization of protein phosphorylation in the soluble protein fraction of scallop (Chlamys farreri) byssus. Mol. Biol. Rep..

[33] Gruffydd LD (1978). The byssus and byssus glands in *Chlamys islandica* and other scallops (*Lamellibranchia*). Zoologica Scr..

[34] Greenwald J, Riek R (2010). Biology of amyloid: structure, function, and regulation. Structure.

[35] Blamires SJ (2015). Mechanical performance of spider silk is robust to nutrient-mediated changes in protein composition. Biomacromolecules.

[36] Gao G, Du G, Sun Y, Fu J (2015). Self-healable, tough, and ultrastretchable nanocomposite hydrogels based on reversible polyacrylamide/montmorillonite adsorption. ACS Appl. Mater. interfaces.

[37] Pasche D (2019). Self-healing silk from the sea: role of helical hierarchical structure in Pinna nobilis byssus mechanics. Soft matter.

[38] Xu Q (2019). Metal coordination-mediated functional grading and self-healing in mussel byssus cuticle. Adv. Sci..

[39] Liu C (2015). Extensible byssus of *Pinctada fucata*: Ca^2+^-stabilized nanocavities and a thrombospondin-1 protein. Sci. Rep..

[40] Negishi A (2012). The production of fibers and films from solubilized hagfish slime thread proteins. Biomacromolecules.

[41] Sun J (2020). Fabrication and mechanical properties of engineered protein-based adhesives and fibers. Adv. Mater..

[42] Giesa T, Schuetz R, Fratzl P, Buehler MJ, Masic A (2017). Unraveling the molecular requirements for macroscopic silk supercontraction. ACS Nano.

[43] Song PG, Xu ZG, Lu Y, Guo QP (2015). Bio-inspired hydrogen-bond cross-link strategy toward strong and tough polymeric materials. Macromolecules.

[44] Van Wart HE, Scheraga HA (2002). Raman spectra of strained disulfides. Effect of rotation about sulfur-sulfur bonds on sulfur-sulfur stretching frequencies. J. Phys. Chem..

[45] Liu FR, Wang L, Wang R, Chen ZX (2013). Calcium-binding capacity of wheat germ protein hydrolysate and characterization of Peptide-calcium complex. J. Agric. Food Chem..

[46] Zhang ZH, Han Z, Zeng XA, Wang MS (2017). The preparation of Fe-glycine complexes by a novel method (pulsed electric fields). Food Chem..

[47] Zhang W (2018). Tensan silk-inspired hierarchical fibers for smart textile applications. ACS Nano.

[48] Wang H (2020). Laser writing of janus graphene/kevlar textile for intelligent protective clothing. ACS Nano.

[49] Wang H (2020). Bioinspired fluffy fabric with in situ grown carbon nanotubes for ultrasensitive wearable airflow sensor. Adv. Mater..

[50] Li S (2019). Physical sensors for skin‐inspired electronics. Infomat.

[51] Liang X (2020). Stable and biocompatible carbon nanotube ink mediated by silk protein for printed electronics. Adv. Mater..

[52] Wang C, Xia K, Zhang Y, Kaplan DL (2019). Silk-based advanced materials for soft electronics. Acc. Chem. Res.

[53] Ye C (2019). Design and fabrication of silk templated electronic yarns and applications in multifunctional textiles. Matter.

[54] Liu M (2021). Biomimicking antibacterial opto-electro sensing sutures made of regenerated silk proteins. Adv. Mater..

[55] Brennan B (2017). Structural, chemical and electrical characterisation of conductive graphene-polymer composite films. Appl. Surf. Sci..

[56] Wang F (2017). Tunable green graphene-silk biomaterials: Mechanism of protein-based nanocomposites. Mater. Sci. Eng. C., Mater. Biol. Appl..

[57] Malard LM, Pimenta MA, Dresselhaus G, Dresselhaus MS (2009). Raman spectroscopy in graphene. Phys. Rep..

[58] Wang JT (2014). Directly obtaining high strength silk fiber from silkworm by feeding carbon nanotubes. Mater. Sci. Eng. C.-Mater. Biol. Appl..

[59] Hou J (2018). Carbon-nanotube-wrapped spider silks for directed cardiomyocyte growth and electrophysiological detection. ACS Appl. Mater. interfaces.

[60] Chi N, Wang R (2018). Electrospun protein-CNT composite fibers and the application in fibroblast stimulation. Biochem. Biophys. Res. Commun..

[61] Yin Z (2018). Splash-resistant and light-weight silk-sheathed wires for textile electronics. Nano Lett..

[62] Zhang X (2018). Characterization of an atypical metalloproteinase inhibitors like protein (Sbp8-1) from scallop byssus. Front. Physiol..

