# Supplementary material for: Extensible and self-recoverable proteinaceous materials derived from scallop byssal thread
Source: Nat Commun. 2022 May 18;13:2731. doi: 10.1038/s41467-022-30415-3 (PMC9117251; doi:10.1038/s41467-022-30415-3)
Supplement: Supplementary file 1 — Supplementary Information [file 41467_2022_30415_MOESM1_ESM.pdf]

Supplementary Information

Extensible and Self-recoverable Proteinaceous Materials Derived from Scallop Byssal Thread

Xiaokang Zhang[1,2#], Mengkui Cui[3#], Shuoshuo Wang[1,2#], Fei Han[4#], Pingping Xu[1,2], Luyao Teng[1,2], Hang Zhao[4], Ping Wang[4], Guichu Yue[5], Yong Zhao[5], Guangfeng Liu[6], Ke Li[3], Jicong Zhang[3], Xiaoping Liang[7], Yingying Zhang[7], Zhiyuan Liu[4*], Chao Zhong[8,9*], Weizhi Liu[1,2*]

[1]*Sars-Fang Centre, MOE Key Laboratory of Marine Genetics and Breeding, College of Marine Life Sciences, Ocean University of China, Qingdao 266003, China.*
[2]*Laboratory for Marine Biology and Biotechnology, Pilot National Laboratory for Marine Science and Technology, Qingdao, 266071, China.*
[3]*Materials and Physical Biology Division, School of physical Science and Technology, ShanghaiTech University, Shanghai 201210, China.*
[4]*Neural Engineering Centre, Shenzhen Institute of Advanced Technology, Chinese Academy of Sciences, Shenzhen 518055, China.*
[5]*School of Chemistry, Beihang University, Beijing 100191, China.*
[6]*National Center for Protein Science Shanghai, Shanghai Advanced Research Institute, Chinese Academy of Sciences, Shanghai 201204, China.*
[7]*Key Laboratory of Organic Optoelectronics and Molecular Engineering of the Ministry of Education, Department of Chemistry and Center for Nano and Micro Mechanics, Tsinghua University, Beijing 100084, PR China.*
[8]*Center for Materials Synthetic Biology, Shenzhen Institute of Synthetic Biology, Shenzhen Institutes of Advanced Technology, Chinese Academy of Sciences, Shenzhen 518055, China.*
[9]*CAS Key Laboratory of Quantitative Engineering Biology, Shenzhen Institute of Synthetic Biology, Shenzhen Institutes of Advanced Technology, Chinese Academy of Sciences, Shenzhen 518055, China.*

[#]These authors contributed equally to this work.

[*]Corresponding authors:

Email: zy.liu1@siat.ac.cn; chao.zhong@siat.ac.cn; liuweizhi@ouc.edu.cn

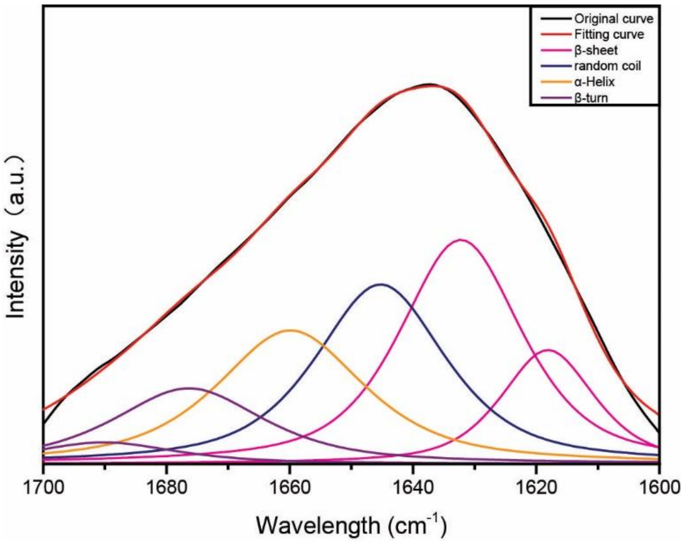

Supplementary Figure 1. The FTIR amide I band and the curve-fitting results of scallop byssal thread.

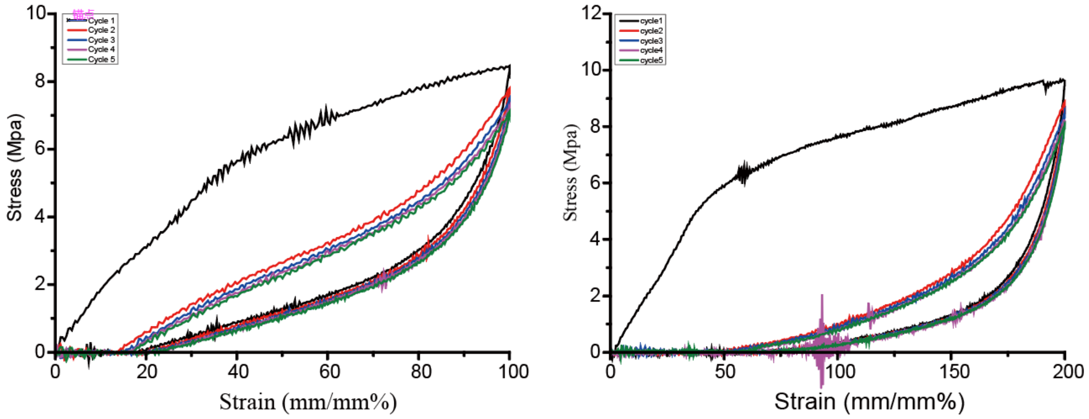

Supplementary Figure 2. Cyclic tensile test of scallop byssal thread.

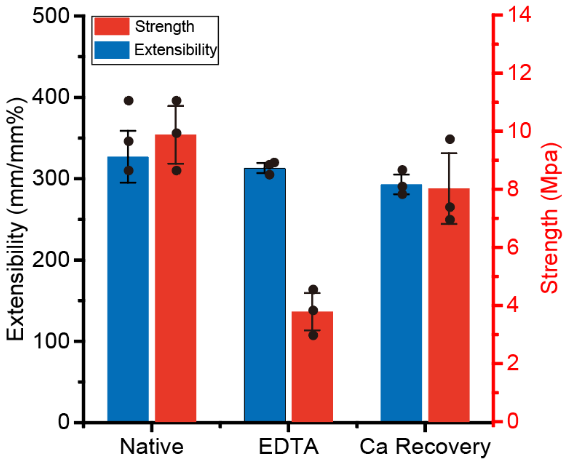

Supplementary Figure 3. Comparison of mechanical properties of scallop byssal thread.

Data are presented as mean values ± SEM. n=3 biologically independent experiments.

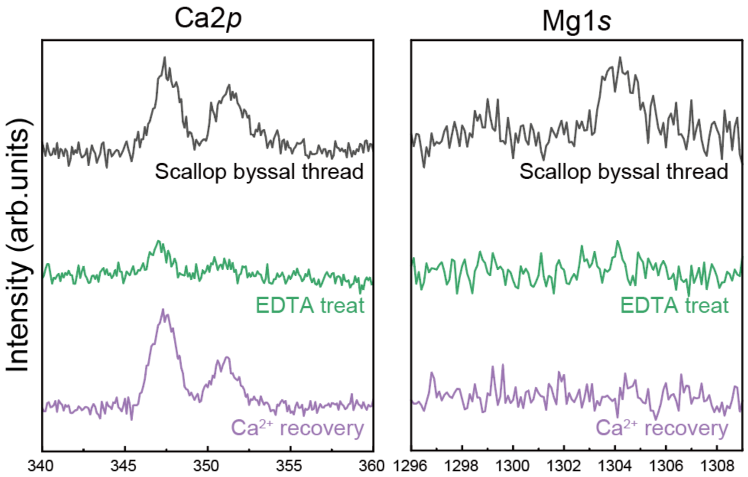

Supplementary Figure 4. XPS analysis of scallop byssal thread.

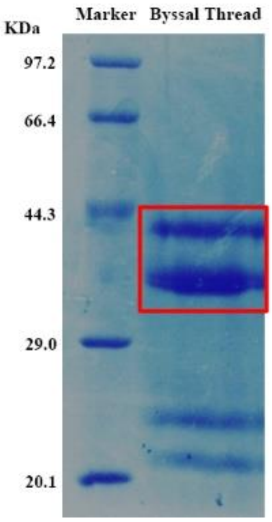

Supplementary Figure 5. Proteins extracted from scallop byssal thread. The red rectangle reprents the main bands used for mass spectrometry.

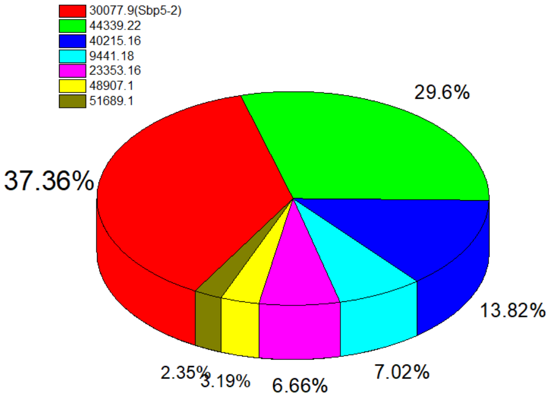

Supplementary Figure 6. Protein abundance analysis for extracted protein from byssal thread based on LFQ intensity of mass spectrometry.

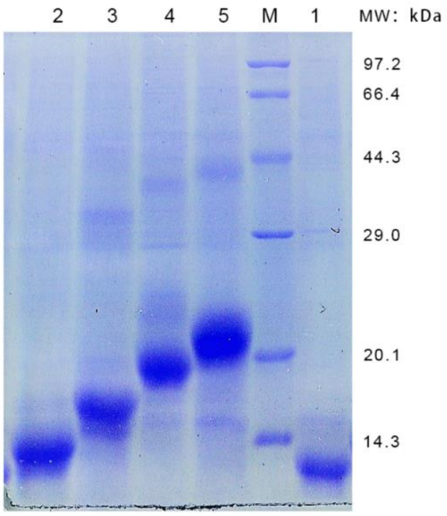

Supplementary Figure 7. SDS-PAGE of purified Sbp5-2 protein with different number of modules.

M, Molecular weight markers; 1, rTRM3; 2, rTRM4; 3, rTRM5; 4, rTRM6; 5, rTRM7.

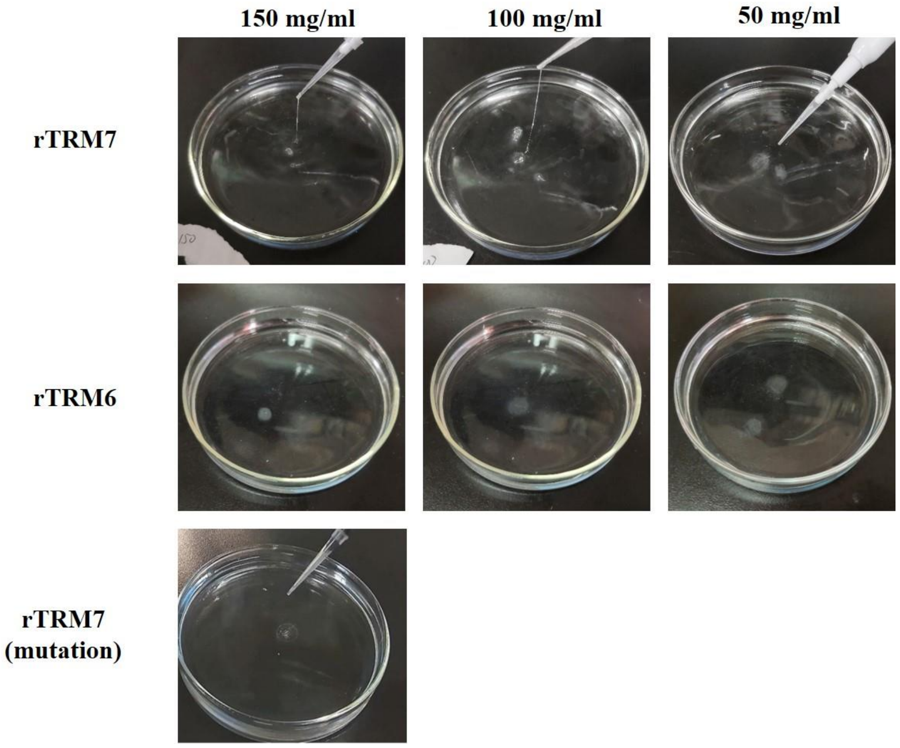

Supplementary Figure 8. Filamentous results of rTRM7 and rTRM6 protein in different concentrations and rTRM7 mutated protein with all Cys mutated to Ser.

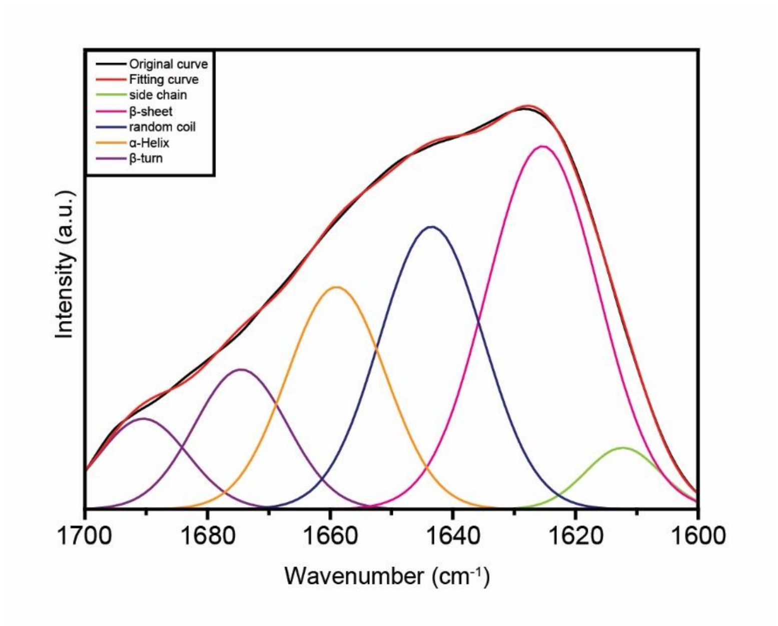

Supplementary Figure 9. The FTIR amide I band and the curve-fitting results of rTRM7.

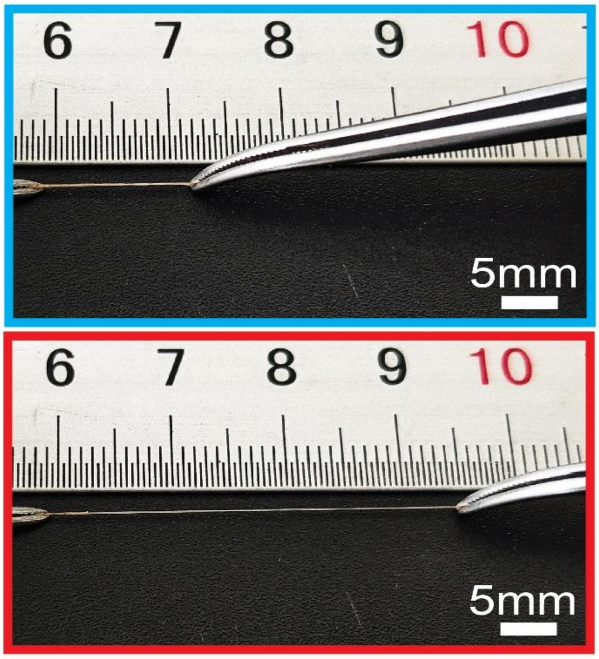

Supplementary Figure 10. Images of rTRM7 fiber before (blue box) and after (red box) stretching with forceps.

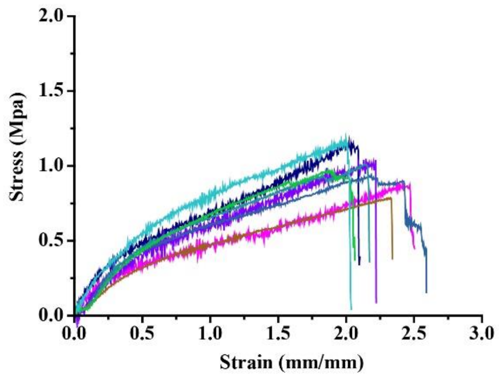

Supplementary Figure 11. Strain-Stress curves of rTRM7 fibers fabricated by hand picking.

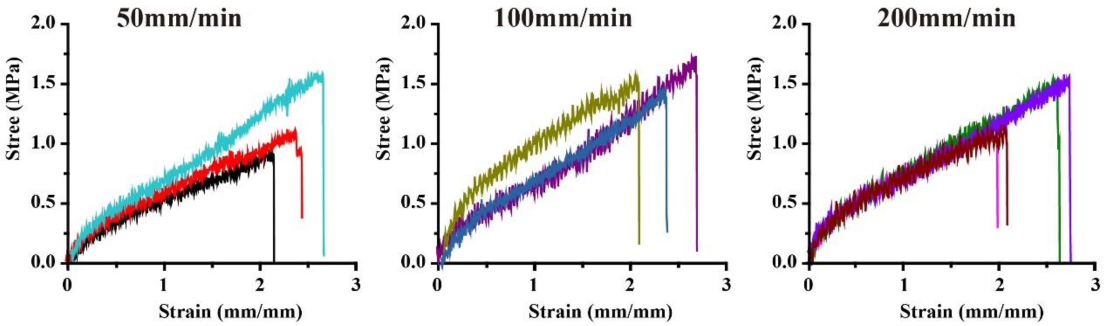

Supplementary Figure 12. Strain-Stress curves of rTRM7 fibers fabricated at different picking speed.

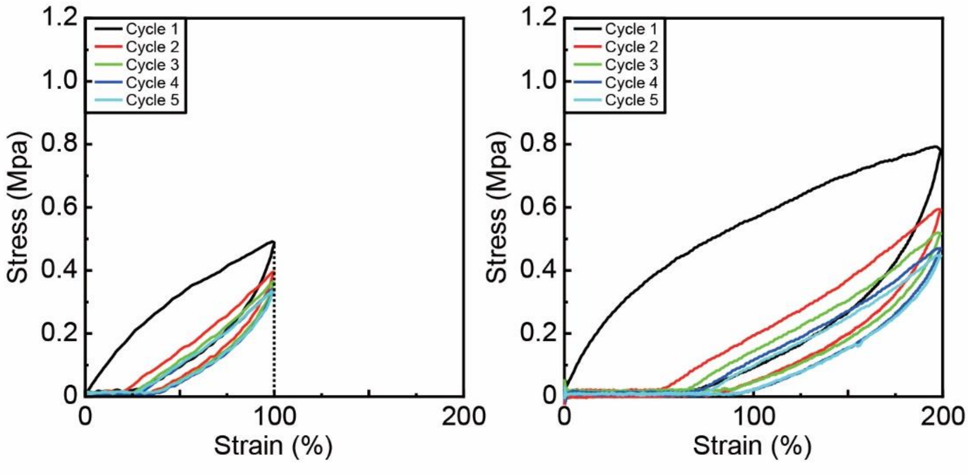

Supplementary Figure 13 Cyclic tensile test of rTRM7 fiber at maximum tensile strain of 100% (left) and 200% (right).

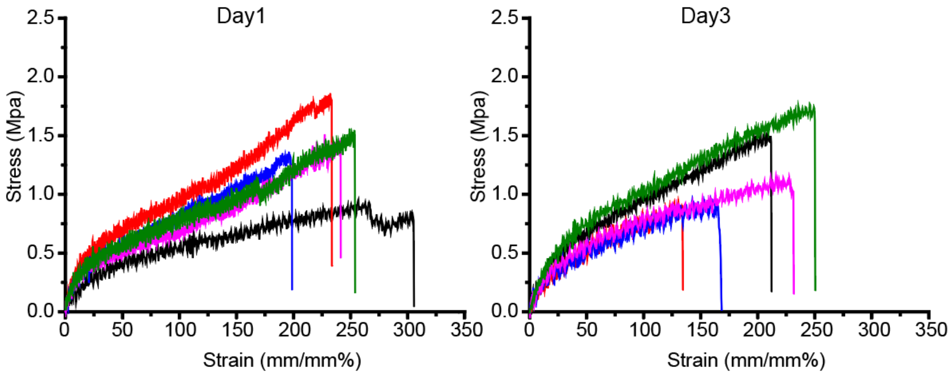

Supplementary Figure 14. Strain-Stress curves of rTRM7 fibers in PBS buffer for 3 days.

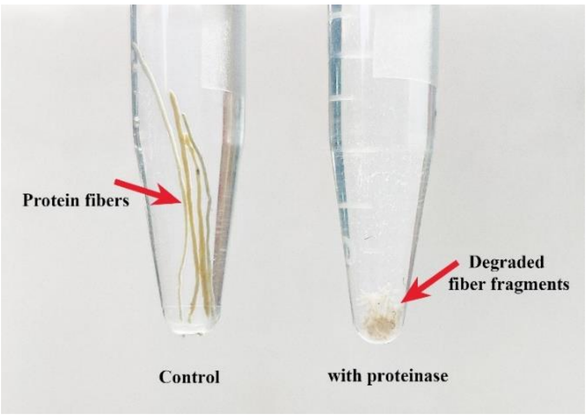

Supplementary Figure 15. Enzymatic degradation of recombinant protein filaments.

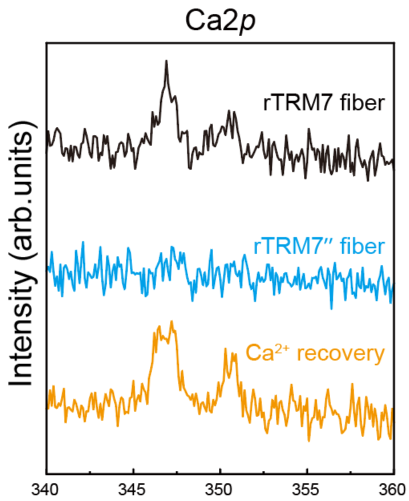

Supplementary Figure 16. XPS analysis of rTRM7 and rTRM7" fibers**.**

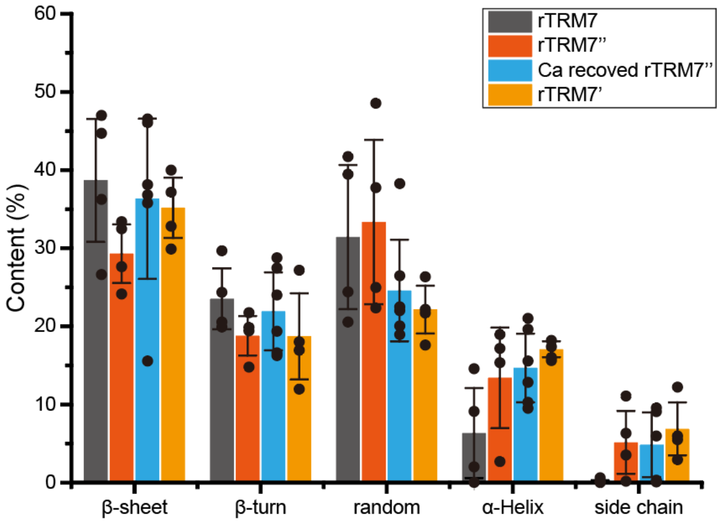

Supplementary Figure 17. Comparison of secondary structure content of rTRM7 thread after different treatments.

Data are presented as mean values ± SEM. n=4 biologically independent experiments.

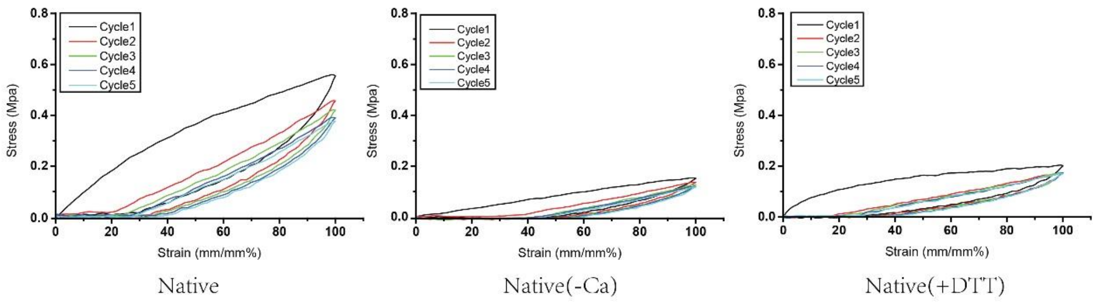

Supplementary Figure 18. Cyclic tensile test of rTRM7, rTRM7' and rTRM7" fibers.

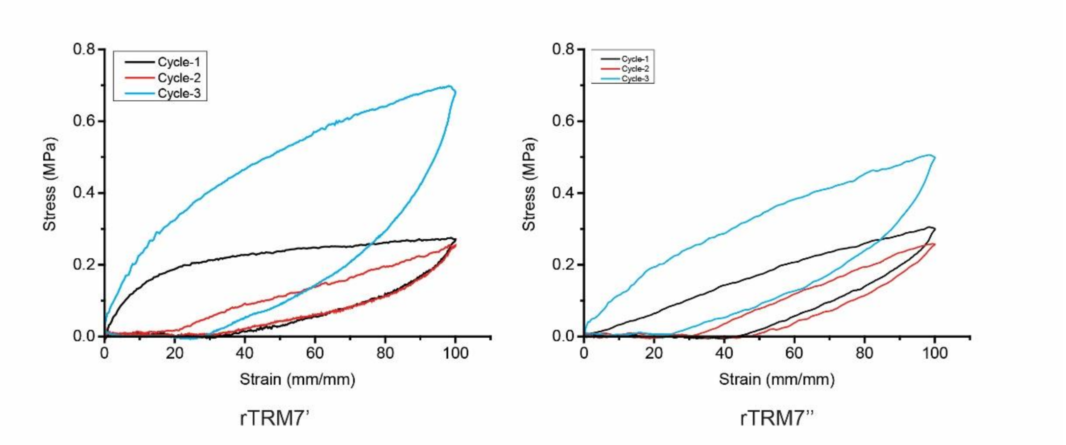

Supplementary Figure 19. Self-recovery test of rTRM7' fiber and rTRM7" fiber.

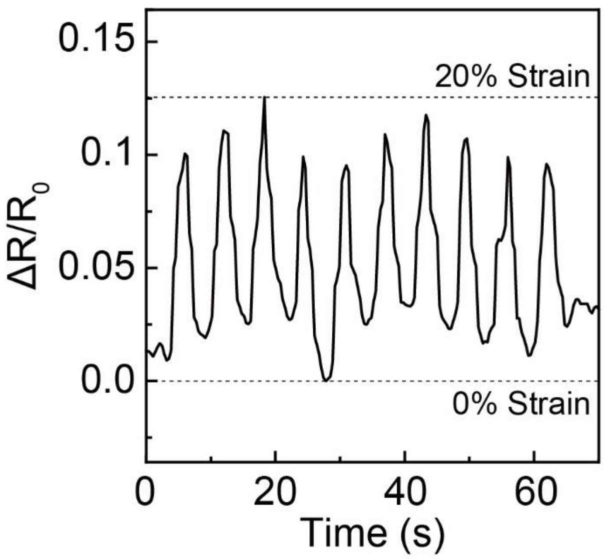

Supplementary Figure 20. Resistance change of rTRM7 fiber over time under cycle loading-unloading stretching.

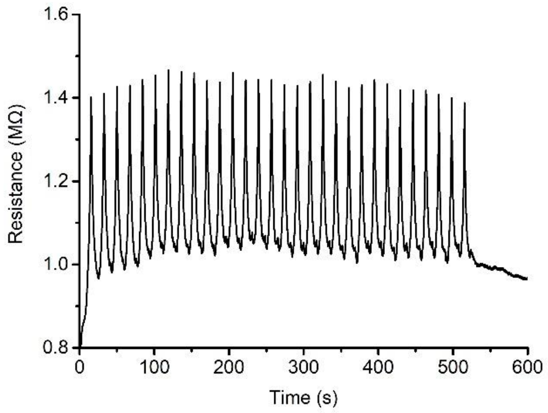

Supplementary Figure 21. The resistance curve under cycle loading-unloading stretching of e-rTRM7.

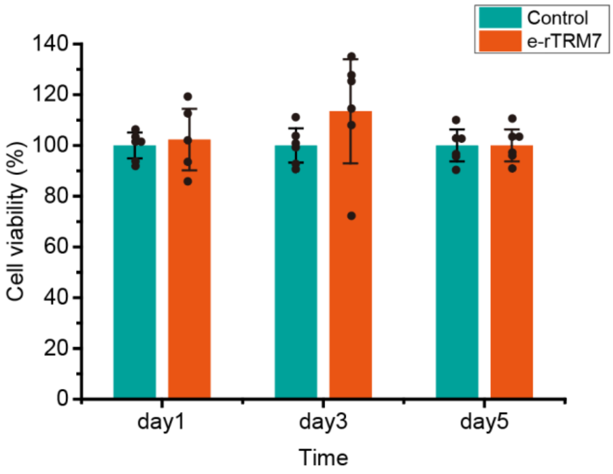

Supplementary Figure 22. In vitro cell viability of e-rTRM7 fiber for L929 cells. Data are presented as mean values ± SEM. n= 6 cells examined over 3 independent experiments.

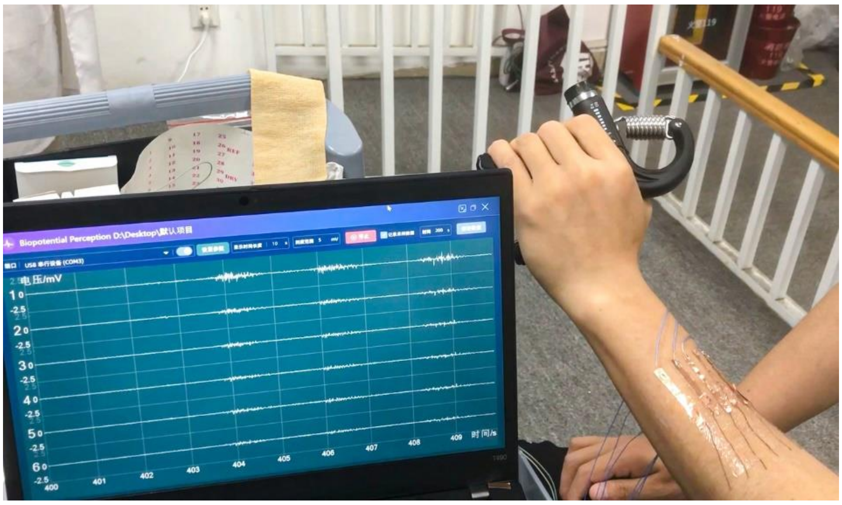

Supplementary Figure 23. Six channels skin EMG signals monitoring when placed on the forearm.

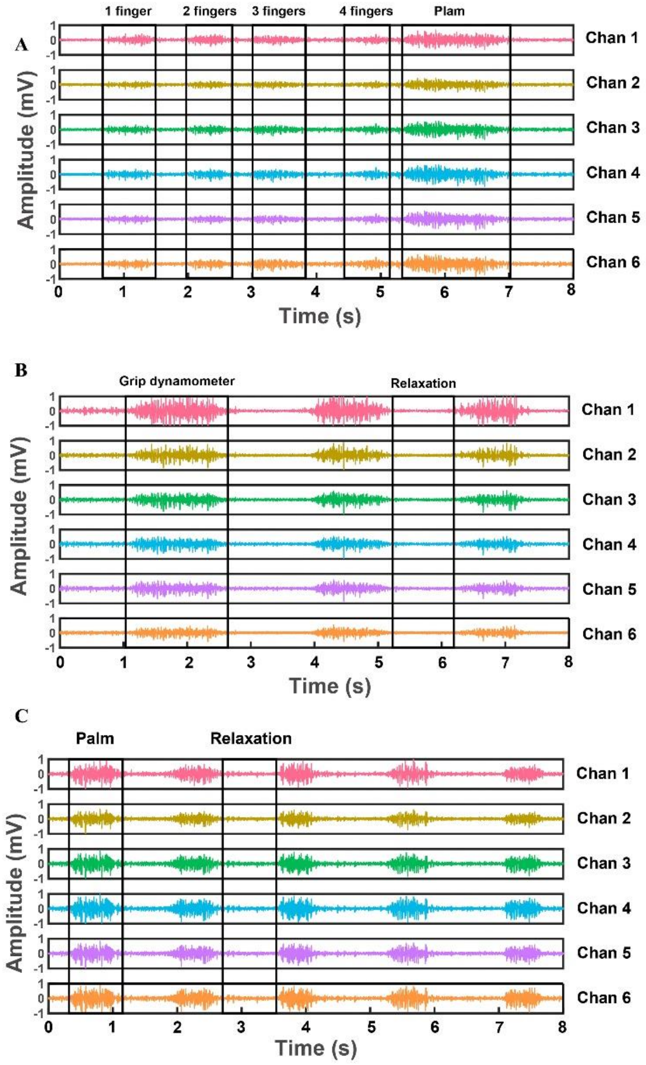

Supplementary Figure 24. Skin electromyography (EMG) results under different movements.

Different numbers of fingers sticking out states (A), Grip the dynamometer and relaxation state (B), Hand palm and relaxation state (C).

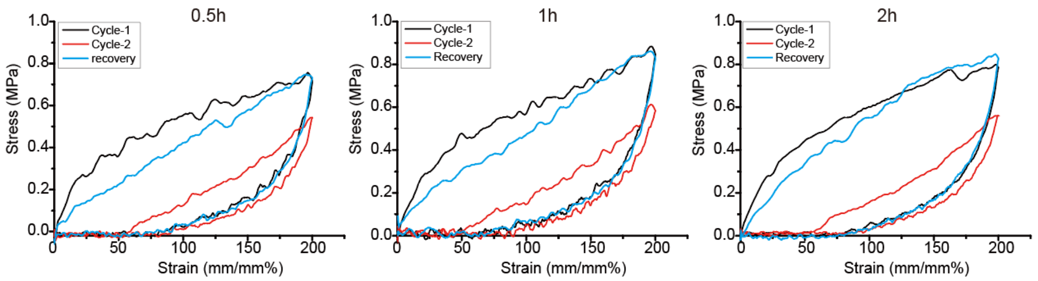

Supplementary Figure 25. Self-recovery test of rTRM7 fiber under different healing time.

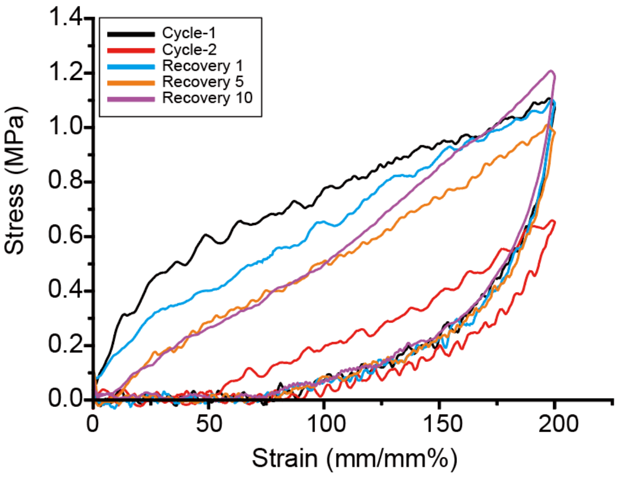

Supplementary Figure 26. Self-recovery test of rTRM7 fiber. Cycle-1 and cycle-2, the first and second stretch cycle without incubation; Recovery 1, Recovery 5 and Recovery 10, the representative stretch cycles of the protein fiber after incubation.

Supplementary Table 1. Amino acid content in byssal thread of *Chlamys farreri*

Amino acids	Content (mg)	Content (%)
Asp	1.571	11.44
Thr	0.813	5.92
Ser	0.708	5.16
Glu	0.995	7.25
Gly	0.923	6.72
Ala	0.417	3.03
Cys	0.379	2.76
Val	0.791	5.76
Met	0.005	0.04
Ile	0.541	3.94
Leu	0.419	3.05
Tyr	0.463	3.37
Phe	0.474	3.45
His	0.594	4.33
Lys	0.783	5.7
Arg	0.53	3.86
Pro	0.839	6.11
**Total**	**11.245**	**81.89**

Supplementary Table 2. Statistics of the relative content of secondary structures of scallop byssal thread (%)

	β-sheet	random	α-Helix	β-turn
Byssal thread	38.83 ± 1.75	26.55 ± 2.33	20.98 ± 1.45	13.64 ± 2.07

Supplementary Table 3. Statistics of mechanical properties of scallop byssal thread

	Native byssal thread	EDTA treated	Ca Recovered
Strength（MPa）	9.88 ± 0.99	3.79 ± 0.65	8.03 ± 1.22
Extensibility(mm/mm%)	327 ± 32	313 ± 6	293 ± 12

Supplementary Table 4. RACE primers of Sbp5-2

	3'-primer	5'-primer
Sbp-5-2-RACE	TCCCAACAATGGAGTATGTGAAGATGCTG	GCCTAGTGTAACAGTCATTCTCCAGCCAAG
rTRM7	CCGCTCGAGTTACGGACTTTTACACACTGGT	GGGGTACCAACAGTTGCACCTCATATATGTGTG
rTRM6	CCGCTCGAGTTACGGACTTTTACACACTGGT	GGGGTACCACAGGTTGTAAAACGGACGAAG
rTRM5	CCGCTCGAGTTACGGACTTTTACACACTGGT	GGGGTACCTCTTCCTGTGTTACCAGACG
rTRM4	CCGCTCGAGTTACGGACTTTTACACACTGGT	GGGGTACCCATGGATGCCGTGCTTCCAACGGT
rTRM3	CCGCTCGAGTTACGGACTTTTACACACTGGT	GGGGTACCAATGACTGTTACACTAGGCG

Supplementary Table 5. Statistics of secondary structure area of rTRM7 thread (%)

	Side Chain	β-sheet	random	α-Helix	β-turn
rTRM7	0.12±0.20	38.69±7.86	31.43±9.21	6.33±5.77	23.53±3.89
rTRM7'	6.88±3.39	35.18±3.86	22.16±3.06	17.06±1.02	18.73±5.51
rTRM7''	5.14±4.02	29.31±3.74	33.35±10.52	13.42±6.44	18.79±2.53
Ca Recovered rTRM7''	4.85±4.14	36.34±10.25	24.57±6.51	14.68±4.39	21.93±4.99

Reference

1 Miao, Y. *et al.* Integration of transcriptomic and proteomic approaches provides a core set of genes for understanding of scallop attachment. *Mar Biotechnol (NY)* **17**, 523-532, doi:10.1007/s10126-015-9635-y (2015).

2 Zhang, X. *et al.* Characterization of an Atypical Metalloproteinase Inhibitors Like Protein (Sbp8-1) From Scallop Byssus. *Frontiers in Physiology* **9**, doi:10.3389/fphys.2018.00597 (2018).
