# Supplementary material for: Extensible and self-recoverable proteinaceous materials derived from scallop byssal thread
Source: Nat Commun. 2022 May 18;13:2731. doi: 10.1038/s41467-022-30415-3 (PMC9117251; doi:10.1038/s41467-022-30415-3)
Supplement: Supplementary file 2 — Description of Additional Supplementary Files [file 41467_2022_30415_MOESM2_ESM.docx]

**Description of Additional Supplementary Files**

**Supplementary Movie 1:** Tensile test of scallop byssal thread.

**Supplementary Movie 2:** Tensile test of recombinant protein fiber.

**Supplementary Movie 3:** Skin electromyography (EMG) test under different movements.

**Supplementary Movie 4:** In vivo action potential signal test of tibialis anterior muscle and tibial nerve.

**Supplementary Movie 5:** Demonstration of preparation of recombinant protein fiber.

**Supplementary Movie 6:** The recombinant protein fibers were prepared by dip coater at different picking speeds.
